# A high-throughput screening identifies histone deacetylase inhibitors as therapeutic agents against medulloblastoma

**DOI:** 10.1186/s40164-019-0153-x

**Published:** 2019-11-15

**Authors:** Shanshan Zhang, Zhaojian Gong, Peter O. Oladimeji, Duane G. Currier, Qipan Deng, Ming Liu, Taosheng Chen, Yong Li

**Affiliations:** 1 Section of Epidemiology & Population Sciences, Department of Medicine, Baylor College of Medicine, Houston, TX USA; 2 Department of Cancer Biology, Lerner Research Institute, Cleveland Clinic, Cleveland, OH USA; 3 Department of Stomatology, Xiangya Hospital, Central South University, Changsha, China; 4 Department of Stomatology, The Second Xiangya Hospital, Central South University, Changsha, China; 5 Department of Chemical Biology and Therapeutics, St. Jude Children’s Research Hospital, Memphis, TN USA; 6 State Key Laboratory of Respiratory Diseases, Guangzhou Institute of Respiratory Diseases, The First Affiliated Hospital of Guangzhou Medical University, Guangzhou Medical University, Guangzhou, China

**Keywords:** High-throughput screening, Medulloblastoma, HDAC inhibitors, Dacinostat, Quisinostat

## Abstract

**Background:**

Medulloblastoma is the most frequently occurring malignant brain tumor in children. Current treatment strategies for medulloblastoma include aggressive surgery, cranio-spinal irradiation and adjuvant chemotherapy. Because current treatments can cause severe long-term side effects and are not curative, successful treatment remains a challenge.

**Methods:**

In this study, we employed a high-throughput cell viability assay to screen 12,800 compounds and to identify drug candidates with anti-proliferative properties for medulloblastoma cells. We also tested these compounds for attenuating medulloblastoma tumor development using mouse xenografts.

**Results:**

We identified two histone deacetylase inhibitors (dacinostat and quisinostat) with anti-proliferative properties for medulloblastoma cells. We showed that both compounds induce cytotoxicity, trigger cell apoptosis, and block cell cycle progression at the G2/M phase. In addition, dacinostat and quisinostat attenuated xenograft medulloblastoma growth in mice.

**Conclusions:**

Our findings suggest that histone deacetylase inhibitors are potent therapeutic agents against medulloblastoma.

## Introduction

Medulloblastoma is the most common malignant pediatric brain tumor, accounting for nearly 20% of all childhood central nervous system malignancies [1]. More than 80% of medulloblastoma are diagnosed in children who are younger than 15 years of age. At least four distinct subtypes comprise medulloblastoma: WNT-activated, SHH-activated, non-WNT/non-SHH group 3, and non-WNT/non-SHH group 4, with group 3 patients carrying c-Myc overexpression having the worst prognosis, as reflected in the current revision of the WHO classification [2–5]. Current treatment strategies for medulloblastoma include aggressive surgery, cranio-spinal irradiation, and adjuvant chemotherapy, with the strategy chosen depending on whether disease is high-risk or low-risk. Successful treatment of medulloblastoma remains a challenge in many patients. Although the 5-year overall survival rate for children with average-risk disease is 70% to 80%, most patients suffer from therapy-related side effects [6]. New treatment strategies are needed that improve patient survival and have fewer adverse effects.

Histone deacetylases (HDACs) are critical epigenetic regulators that have been implicated in oncogenesis because they can silence tumor suppressor genes and genes that induce apoptosis [7, 8]. As HDACs are frequently upregulated in cancers, they represent potential therapeutic targets. HDAC inhibitors are a promising group of anti-cancer drugs that change the gene expression patterns of cancer cells by epigenetic modulation. In this study, we performed a high-throughput drug screening to identify compounds that inhibit medulloblastoma growth and tumorigenesis. We found that two HDAC inhibitors, dacinostat and quisinostat, are superior to other compounds in elevating medulloblastoma apoptosis and attenuating tumor growth. Strikingly, both compounds reduced the expression of c-Myc. Given that HDAC expression has been reported to be upregulated in medulloblastoma [9], these data suggest that HDAC inhibitors are a new strategy to treat this devastating malignancy, particularly group 3 disease with c-Myc overexpression.

## Materials and methods

### Cell lines

The human medulloblastoma cell lines Daoy and D283 were obtained from the American Type Culture Collection (ATCC, Manassas, VA) and cultured in Eagle’s Minimum Essential Medium (MEM) containing 10% fetal bovine serum (Thermofisher, Waltham, MA). Mycoplasma testing was conducted every 3 months to ensure no contamination. All cells were maintained in a humidified incubator at 37 °C and 5% CO_2_. For all studies, medulloblastoma cells were grown to 50–70% confluence on 10-cm plates and then treated with dacinostat and quisinostat for the indicated time periods. All methods related to human cells were carried out in accordance with National Institutes of Health (NIH) guidelines and regulations and Cleveland Clinic Institutional Biosafety Committee polices.

### Compound screening

We developed a cytotoxicity assay using the CellTiter-Glo Luminescent Cell Viability Assay (Promega, Madison, WI) to screen a bioactive compound library (including FDA-approved drugs) [10, 11] for compounds that inhibit the growth of Daoy cells. Cells were plated at 3000 cells/well in 384-well tissue culture plates. Compounds were added (10 µM for the primary screen and 10 different concentrations as indicated in the dose–response analysis), and cells were incubated at 37 °C and 5% CO_2_ for 48 h. Vehicle (dimethyl sulfoxide [DMSO]: 0.1% for primary screen and 0.5% for dose–response analysis) and staurosporine (10 µM) were used as negative (0% inhibition) and positive (100% inhibition) controls, respectively. From the primary screen of 12,800 compounds, we selected 125 compounds that displayed ≥ 90% inhibition for dose–response analysis (10 concentrations, 1:3 serially diluted: 0.0025, 0.0076, 0.023, 0.068, 0.2, 0.6, 1.9, 5.6, 16.7, and 50 µM). Of the 125 tested, 113 compounds were confirmed to inhibit cell growth in a dose-responsive manner. Examples include pitavastatin, which has an IC_50_ value of 84 nM. The general screening process and data analysis have been previously described [12–15].

### Chemical compounds

Dacinostat and quisinostat were obtained from MedChemExpress LLC (Princeton, NJ). These two compounds were dissolved in DMSO (10 mM/mL) before diluted with phosphate-buffered saline (PBS) to treat cells or mice.

### Cell viability assay

Medulloblastoma cells were seeded in sextuplicate in 96-well plates and treated for 48 h with dacinostat and quisinostat (0, 0.0025, 0.0076, 0.023, 0.068, 0.2, 0.6, 1.9, 5.6, 16.7, or 50 µM). Viability was measured using the MTT Cell Proliferation Assay Kit (ATCC, Manassas, VA).

### Apoptosis and cell cycle distribution assessment

Medulloblastoma cells were incubated with dacinostat or quisinostat at their IC_50_ concentration for 24 h and 48 h. After incubation, floating and adherent cells were collected, washed with serum-free medium, and suspended in PBS. The cell suspension was stained with FITC-conjugated annexin V and propidium iodide (PI) using the Alexa Fluor 488 annexin V/Dead Cell Apoptosis Kit (Thermofisher) and then analyzed by flow cytometry. Cell apoptosis and cell cycle distribution analyses were performed using FlowJo (FlowJo LLC, Ashland, OR).

### Protein extracts and Western blotting analysis

After incubating Daoy and D283 cells with dacinostat or quisinostat for the indicated amount of time, cells were collected and washed with PBS. Cells were lysed with RIPA lysis buffer containing protease and phosphatase inhibitors to extract the soluble cellular proteins. The lysates were boiled for 5 min at 95 °C. Protein concentration was measured with the BCA protein assay reagent (Pierce, Rockford, IL, USA). The samples were diluted with lysis buffer containing 20 mM dithiothreitol, and equal amounts of protein were loaded on 10% to 15% SDS-polyacrylamide gels (Mini-PROTEAN TGX Precast gels, Bio-Rad, Hercules, CA), separated, and transferred onto polyvinylidene difluoride (PVDF) membranes. The membrane was blocked with 5% nonfat dry milk in Tris-buffered saline (TBS) containing 0.1% Tween-20 (v/v) for 1 h and incubated with primary antibody at 4 °C overnight. The primary antibodies were purchased from Cell Signaling Technology (Danvers, MA, USA): caspase-3 (#9662), cleaved-caspase-3 (#9661), cleaved-PARP (#5625), Acetyl-Histone H3 (#8173), acetyl-histone H4 (#13944), and β-actin (#3700). Horseradish peroxidase-conjugated anti-rabbit or anti-mouse IgG was used as the secondary antibody. Immunoreactive proteins were visualized with the Pierce ECL Western blotting substrate, according to the provided protocol. Blots were quantified by scanning densitometry using area integration.

### Animal models

Human medulloblastoma Daoy xenograft tumors were established by subcutaneously injecting 2 × 10^6^ cells suspended in 100 µm PBS into the right flank of 6-week-old male NSG mice (Jackson, MA, USA). Tumors were measured every 3 days using calipers. Tumor volume was calculated as width × length × height × 0.52. When tumor volumes reached 100 mm^3^, treatment was administered by intraperitoneal injection every 2 days. Mice were randomized into four treatment groups (n = 5 per group). Both dacinostat and quisinostat diluted in PBS were administered at 20 mg/kg body weight. DMSO diluted in proper volume of PBS was used as the vehicle control. Mice were fed ad libitum and maintained in environments with a controlled temperature of ~ 22 °C and 12 h light and dark cycles. After 18 days of treatment, animals were sacrificed, and tumors were excised and analyzed. All procedures involving animals were carried out in accordance with NIH guidelines and regulations, and experimental protocols were approved by our Institutional Animal Care and Use Committee.

### Histology and immunohistochemical analyses

For each subcutaneous tumor, 4-µm thick paraffin sections were prepared and stained with hematoxylin and eosin. Immunohistochemistry of Ki-67 and cleaved PARP was performed by the Human Tissue Acquisition and Pathology Core at Baylor College of Medicine. Images from sections were recorded with an Olympus microscope and advanced image acquisition software (version 1.13, cellSens Dimension).

### Statistical analysis

Significant differences between the vehicle controls versus treatment groups were determined by an unpaired two-sided t-test. Statistical analysis was carried out using GraphPad InStat 7 software (GraphPad Software, Inc., San Diego, CA, USA). P ≤ 0.05 was considered statistically significant.

## Results

### High-throughput screening identified dacinostat and quisinostat

**Fig. 1:**
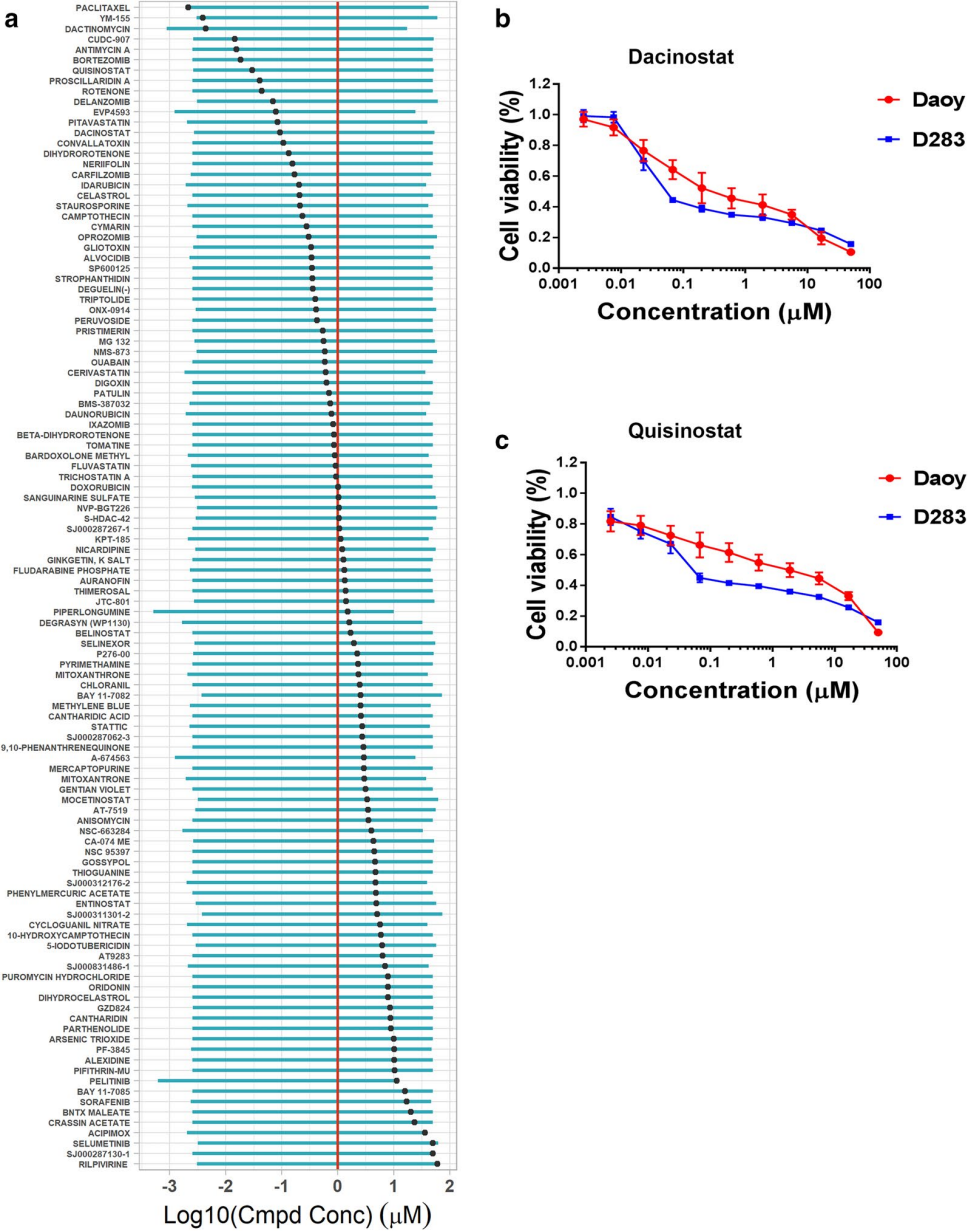
High-throughput screen identifies dacinostat and quisinostat that reduce cell viability of Daoy and D283 cells. **a** 125 compounds were assayed for their IC_50_ values after the screening of 12,800 compounds. Blue bars represent the concentration range interrogated and black points indicate the IC_50_ value derived from the curve fit. The red line represents the 1 μM IC_50_ cutoff used to identify the 46 most potent compounds. Compounds having names too long to fit on the plot axis are identified by their compound identifier; their names are as follows: SJ000831486-1 = BML285 (diaminoquinazoline); SJ000287267-1 = cephaeline dihydrochloride heptahydrate; SJ000311301-2 = NA; SJ000312176-2 = aurora kinase/CDK inhibitor; SJ000287062-3 = 1-benzyloxycarbonylaminophenethyl chloromethyl ketone; SJ000287130-1 = lysyltryptophanyl-lysine acetate. Cmpd Conc, compound concentration. **b**, **c** The dose response curves (based on MTT assays) for cell viability of Daoy and D283 cells after treated with dacinostat and quisinostat for 48 h at the indicated concentrations

In our high-throughput screen, we employed the CellTiter-Glo Luminescent Cell Viability Assay, a homogeneous method of measuring the number of viable cells based on quantitation of ATP, which is an indicator of metabolically active cells. We incubated Daoy cells with each compound at a final concentration 10 µM for 48 h. From 12,800 compounds screened, we selected 125 compounds that displayed ≥ 90% inhibition for further analysis in dose responsive manner (10 concentrations, 1:3 serially diluted ranging from 50 to 0.0025 µM in triplicate) (see Additional file 1: Data S1 and Additional file 2: Figure S1). The Z′-factor, which was used to assess assay performance [16], ranged from 0.50 to 0.85, with an average value of 0.72 (Additional file 2: Figure S1B). We found 113 compounds that inhibited cell growth in a dose-responsive manner with an IC_50_ of ≤ 10 µM at 48 h of incubation. Among them, 46 compounds had an IC_50_ of ≤ 1 µM (Fig. 1 and Additional file 1: Data S1). Next, we shortened this list to 7 compounds based on the following criteria: (1) low reported toxicity; (2) IC_50_ ≤ 100 nM (Additional file 1: Data S1).

To minimize false positives, we ordered 6 of these 7 compounds (EVP4593 is not commercially available) in powder form and tested their activities in another medulloblastoma cell line D283. We determined the IC_50_ values of these 6 compounds against D283 cells. Three compounds quisinostat, dacinostat, and proscillaridin A had IC_50_ < 200 nM for D283 cells. Proscillaridin is a cardiac glycoside, a drug used to treat congestive heart failure and cardiac arrhythmia. As both quisinostat and dacinostat are inhibitors to HDACs (Fig. 1b, c), we chose them for follow-up studies.

### Dacinostat and quisinostat induce apoptosis in medulloblastoma cells

**Fig. 2:**
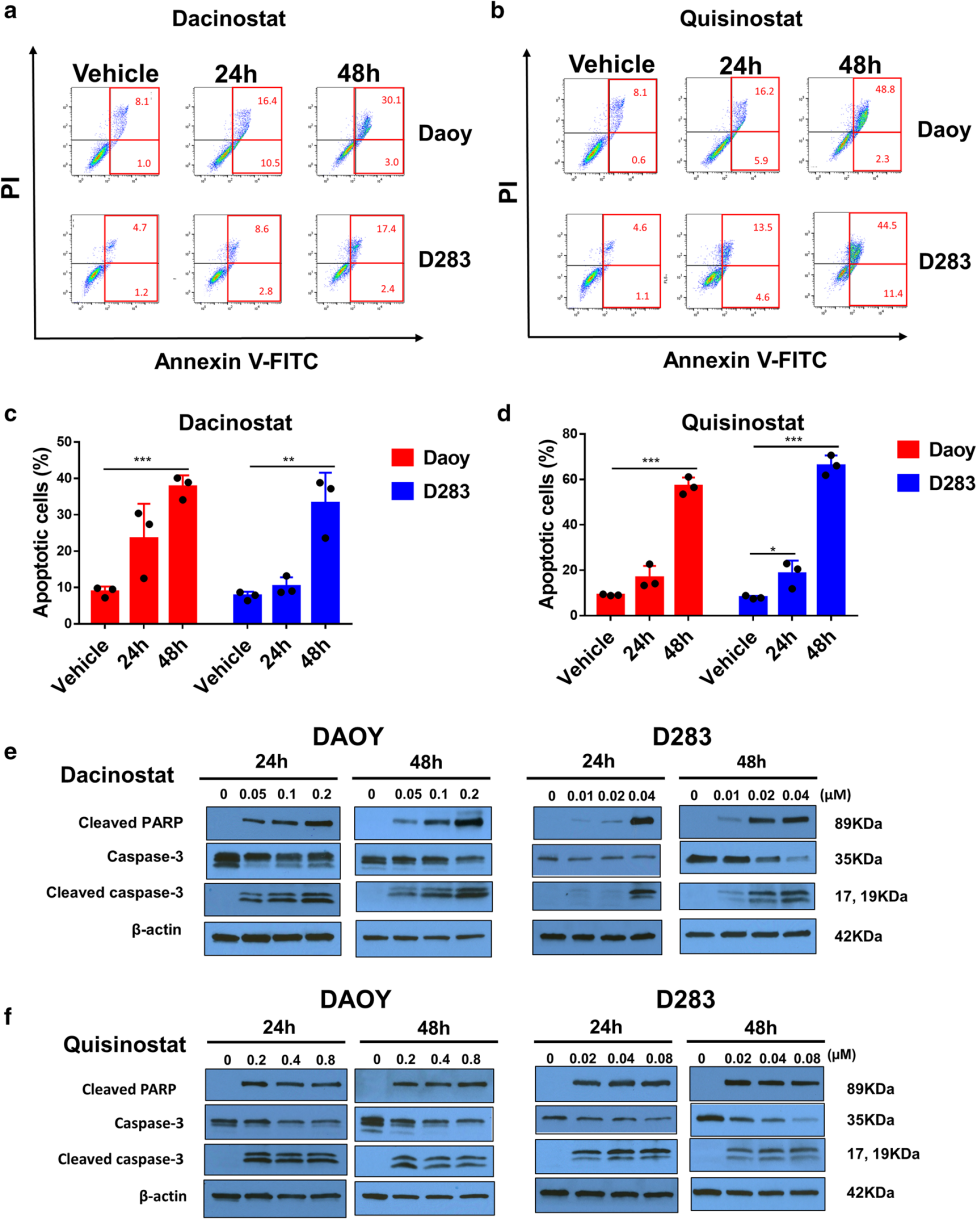
Dacinostat and quisinostat increase apoptosis of Daoy and D283 cells. **a**, **b** Daoy and D283 cells were treated with dacinostat (0.1 µM for Daoy cells, 0.01 µM for D283 cells) or quisinostat (0.4 µM for Daoy cells, 0.04 µM for D283 cells) for 24 h and 48 h, respectively. Apoptosis was analyzed with flow cytometry. Experiments were performed three times with one representative scatterplot shown. **c**, **d** Percentage of apoptotic cells. An unpaired t test was performed to compare each treatment group with the vehicle control (PBS). *P ≤ 0.05, **P ≤ 0.01, ***P ≤ 0.001. **e**, **f** Dacinostat and quisinostat induce the cleavage of caspase-3 and PARP. Daoy and D283 cells were treated with dacinostat and quisinostat at indicated concentrations for 24 h and 48 h, respectively. The target protein expression was analyzed by western blot

We investigated whether dacinostat and quisinostat induced apoptosis in medulloblastoma cells. Both dacinostat and quisinostat treatment resulted in a higher percentage of apoptotic cells, when compared with vehicle group, in a time-dependent manner (Fig. 2a–d). At 48 h, the percentage of apoptotic cells upon treatment with dacinostat increased 24% in Daoy cells and 14% in D283 cells, respectively (Fig. 2c). The percentage of apoptotic cells upon treatment with quisinostat increased 42% in Daoy and 50% in D283, respectively (Fig. 2d). These results suggest that apoptosis induction contributes to the reduced cell viability of both Daoy and D283 cells treated with dacinostat and quisinostat. We next examined the expression of poly-ADP ribose polymerase (PARP) and caspase-3. In treated Daoy and D283 cells, caspase-3 decreased, whereas cleaved caspase-3 and cleaved PARP increased (Fig. 2e, f). These results indicate the increased apoptosis in medulloblastoma cells treated with HDAC inhibitors is mediated by caspase activation.

### Dacinostat and quisinostat induced G2/M arrest of medulloblastoma cells

**Fig. 3:**
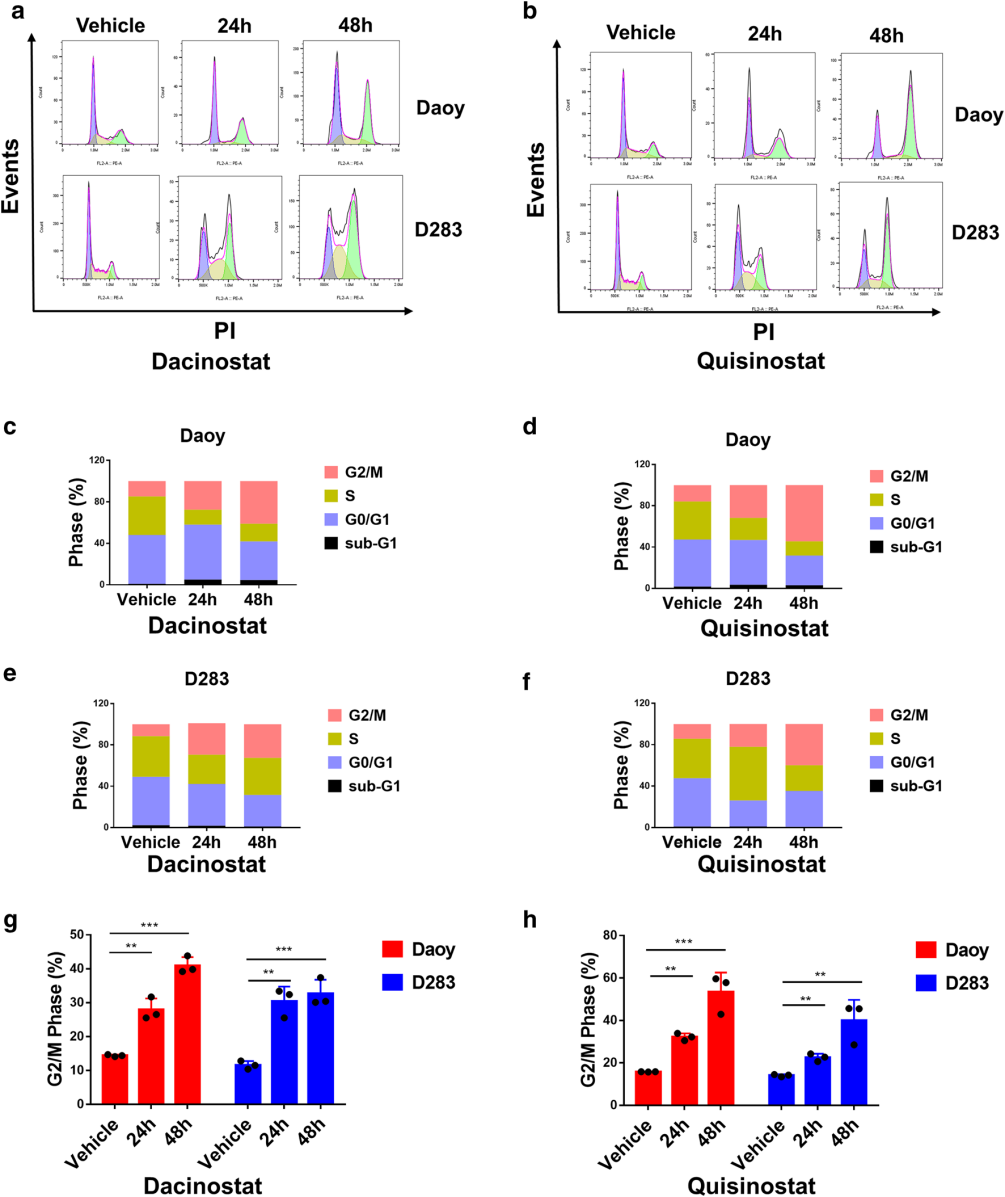
Dacinostat and quisinostat induce G2/M arrest in Daoy and D283 cells. **a**, **b** Daoy and D283 cells were treated with dacinostat (0.1 µM for Daoy cells, 0.01 µM for D283 cells) or dacinostat (0.4 µM for Daoy cells, 0.04 µM for D283 cells) for 24 h and 48 h, respectively. The cell cycle distribution was analyzed by flow cytometry using FlowJo. **c**–**f** Histograms showing the percentage of Daoy and D283 cells in the subG1, G0–G1, S, and G2/M phases. **g**, **h** Percentage of cells in the G2/M phase. *P ≤ 0.05, **P ≤ 0.01, ***P ≤ 0.001. Experiments were performed three times, and values were presented as the mean ± SE

To further characterize the cytotoxic efficacy of dacinostat and quisinostat, we analyzed cell cycle progression. Daoy and D283 cells were treated with dacinostat (0.1 µM for Daoy, 0.01 µM for D283 cells) and quisinostat (0.4 µM for Daoy cells, 0.04 µM for D283 cells) for 24 h and 48 h, subjected them to flow cytometry (Fig. 3a, b), and then determined the percentage of cells in each phase (Fig. 3c–f). The percentage of cells in the G2/M phase significantly increased in both cell lines compared with vehicle treatment (Fig. 3g, h). These data indicate that dacinostat and quisinostat induce cell cycle arrest in medulloblastoma cells.

### Histone acetylation, Akt, and c-Myc expression

**Fig. 4:**
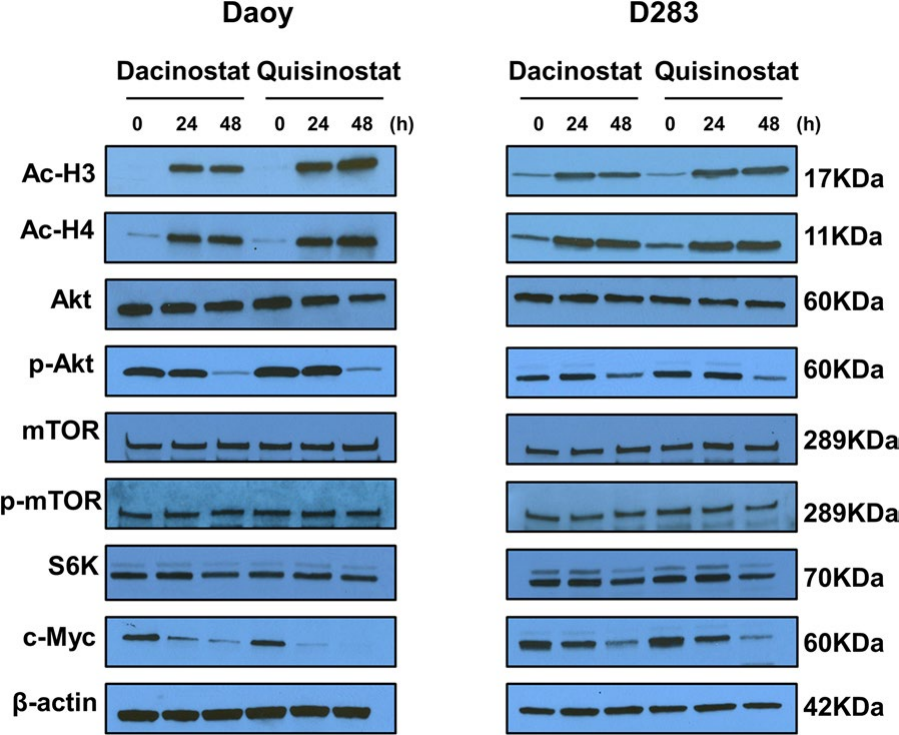
Mechanism of action for compounds. Daoy and D283 cells were treated with dacinostat or quisinostat at their respective IC_50_ concentration (as shown in Additional file 1: Data S1) for 24 h and 48 h. Western blot analysis of Daoy and D283 cells treated with dacinostat and quisinostat

We next performed western blotting analyses to determine histone acetylation and the key cancer pathways associated with HDAC inhibitors (Fig. 4). As expected, the acetylation for both histones H3 and H4 was significantly augmented in treated Daoy and D283 cells. Akt phosphorylation was significantly inhibited at 48 h post-treatment; however, the phosphorylation of mTOR and the expression of S6K were only marginally affected. In agreement with a previous report that HDAC inhibition reduces c-Myc expression, we found that both dacinostat and quisinostat strikingly reduced the expression of c-Myc in Daoy and D283 cells. These data demonstrate that HDAC inhibition elevates histone acetylation and constrain major cancer signaling pathways such as c-Myc and Akt in medulloblastoma cells.

### Dacinostat and quisinostat attenuate Daoy xenograft tumorigenesis

**Fig. 5:**
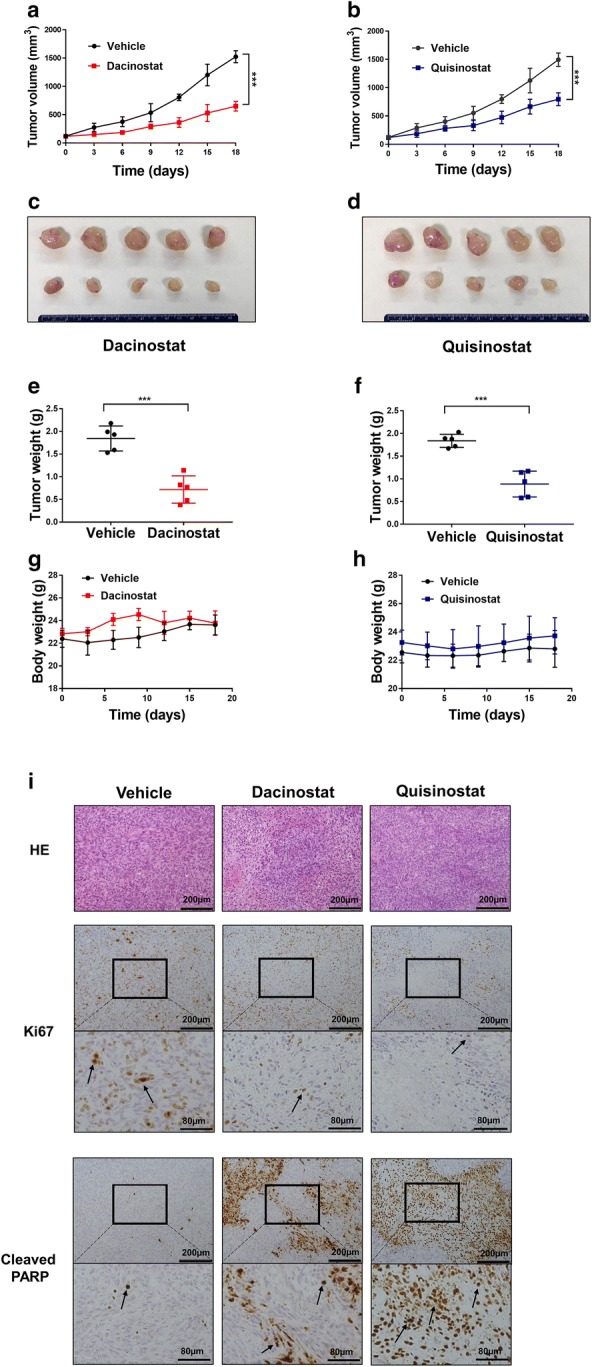
Dacinostat and quisinostat attenuate tumorigenesis of medulloblastoma xenografts in NSG mice. **a**, **b** Tumor volumes during treatment were measured using calipers. Values were presented as the mean ± SE. **c**, **d** Gross tumors at day 18 post-treatment. **e**, **f** Tumor weights at day 18 post-treatment. **g**, **h** Body weight during treatment was measured. Values were presented as the mean ± SE. **i** Histological analysis of tumor. Representative images of immunohistochemistry staining for Ki67 and cleaved PARP in tumor tissues were shown. Black arrows pointed to cells with positive staining. *HE* hematoxylin and eosin staining

Finally, we evaluated the in vivo anti-tumor effects of dacinostat and quisinostat on medulloblastoma xenografts. Daoy cells were inoculated subcutaneously into NSG mice, and once tumors were established, mice were injected intraperitoneally with 20 mg/kg dacinostat or quisinostat every other day. We found that dacinostat or quisinostat treatment suppressed tumor growth significantly (Fig. 5a–f). Animal body weight was not significantly changed by either treatment (Fig. 5g, h). Moreover, dacinostat and quisinostat treatment notably reduced cell proliferation and increased cell apoptosis, as indicated by fewer Ki-67-positive cells and more cleaved PARP-positive cells (Fig. 5i). These data suggest that dacinostat and quisinostat inhibit medulloblastoma tumor cell proliferation, increase apoptosis, and attenuate xenograft tumorigenesis in vivo.

## Discussion

Medulloblastoma represents 12% of childhood brain tumors. Recent advances in cancer genetics and genomics have classified medulloblastoma into four molecular subgroups: WNT, SHH, group 3 (c-Myc overexpression), and group 4. Among them, group 3 patients have the poorest prognosis, as the majority of cases are metastatic at the time of diagnosis [17]. Mocetinostat (MGCD0103), an HDAC1/HDAC2 inhibitor, is found to target Gli1 acetylation to truncate SHH signaling in medulloblastoma cells [18]. Recently, from a 960-compound screening, quisinostat and other class I HDAC inhibitors are found to suppress growth of diverse SHH signaling inhibitor-resistant clones of mouse medulloblastoma cells [19]. For group 3 medulloblastoma, Wechsler-Reya and colleagues have screened 3642 compounds using mouse medulloblastoma cells [20]. They found that HDAC inhibitors were among the agents that inhibited growth of medulloblastoma tumor cells at submicromolar concentrations. Importantly, HDAC inhibitors and PI3K inhibitors cooperate to inhibit the growth of c-Myc-driven mouse medulloblastoma and human patient-derived xenograft tumors [20].

In this study, we employed Daoy cells, a human medulloblastoma cell line resembling the SHH subtype [21], and screened 12,800 compounds for their anti-medulloblastoma activity. We found 46 compounds that inhibited Daoy cell growth in a dose-responsive manner with an IC_50_ of ≤ 1.0 µM for 48 h. In addition, we used D283 cells, a long-established cell line that represents an intermediate subtype between Group 3 and 4 medulloblastoma [21], to further analyze the efficacy of selected compounds. D283 cells show MYC overexpression at the mRNA and protein level and exhibit OTX2 overexpression consistent with Group 3 and 4 [21]. Two compounds, quisinostat and dacinostat (both HDAC inhibitors), significantly inhibited the viability of both Daoy and D238 at submicromolar concentrations. Dacinostat (also known as LAQ824), is a pan-HDAC inhibitor belonging to a class of hydroxamic acid analogs known to inhibit class I, IIa, and IIb histone deacetylases [22, 23]. It has been tested in animal models for its direct antitumor effects, mainly against hematopoietic lineage cancer cells [22, 24–26], but also against various types of solid tumors, such as lung, colon, and prostatic cancers [27–29]. Quisinostat (also known as JNJ26481585), is a second generation pan-HDAC inhibitor. It is effective against several tumor types, including colon cancer [30], glioblastoma [31], leukemia [32], and multiple myeloma [33, 34]. To date, four HDAC inhibitors (panobinostat, romidepsin, heliostat, and vorinostat) have been approved by the United States Food and Drug Administration for the treatment of hematological malignancies, such as cutaneous T-cell lymphoma, peripheral T-cell lymphoma, and multiple myeloma [35–39].

HDACs catalyze the removal of acetyl groups from lysine residues of nuclear histones as well as cytoplasmic substrates, and HDAC inhibition affects diverse cellular processes including cell cycle control and apoptosis [40–43]. We demonstrated that both dacinostat and quisinostat induce cell apoptosis and G2/M arrest in medulloblastoma. Daoy and D283 cells and attenuate xenograft tumorigenesis in immunodeficient mice. Dacinostat and quisinostat exercise their anti-medulloblastoma activity via induction of caspase-3 and PARP cleavage and augmenting the acetylation for histones H3 and H4. Further studies using more cell lines and the orthotopic model will help to move HDAC inhibitors into clinical care for medulloblastoma patients. As Daoy and D283 cells represent different medulloblastoma subtypes, these data support dacinostat and quisinostat as potential drug candidates for broad medulloblastoma therapy.

## Conclusions

Our work shows that dacinostat and quisinostat exhibit effective anti-tumor activity for two different medulloblastoma subtypes in vitro and medulloblastoma mouse xenografts in vivo. Our data call for new clinical trials to evaluate the efficacy of dacinostat, quisinostat, and other HDAC inhibitors, against medulloblastoma.

## Supplementary information

**Additional file 1: Data S1.** Dose response curves of Daoy cells treated with 125 compounds.

**Additional file 2: Figure S1.** Compound activity and plate Z′-factor for the primary screening. (**A**) Compound activity distribution. *Blue dots (Inhibitor Control)*: positive control group (10 μM staurosporine, 100% inhibition); *black dots (Neutral Control)*: negative control group (DMSO group, 0% inhibition); *yellow dots (Hit Compound)*: primary hits (hit compound) (125 compounds that displayed ≥ 90% inhibition chosen for a dose-response analysis); *grey dots (Compound):* compounds not chosen for further confirmation (% inhibition < 90%). Y-axis: % Activity is assessed as the percentage of viability inhibited. The activity from 12,800 library compounds and control compounds in each plate were shown. (**B**) Z′-factor calculated for each plate. (**C**) The dose response curves for cell viability of Daoy cells. Ten concentrations, 1:3 serially diluted ranging from 50 to 0.0025 μM, were used in triplicate.

## Data Availability

All data and materials supporting the conclusion of this study have been included within the article and Additional files.

## Abbreviations

HDAC: histone deacetylase
FDA: Food and Drug Administration
DMSO: dimethyl sulfoxide
PBS: phosphate-buffered saline
ATCC: American Type Culture Collection
NIH: National Institutes of Health
PI: propidium iodide
NSG: NOD scid gamma
WNT: wingless-related integration site
SHH: sonic hedgehog
