# Supplementary figures and images for: A high-throughput screening identifies histone deacetylase inhibitors as therapeutic agents against medulloblastoma
Source: Exp Hematol Oncol. 2019 Nov 15;8:30. doi: 10.1186/s40164-019-0153-x (PMC6858705; doi:10.1186/s40164-019-0153-x)

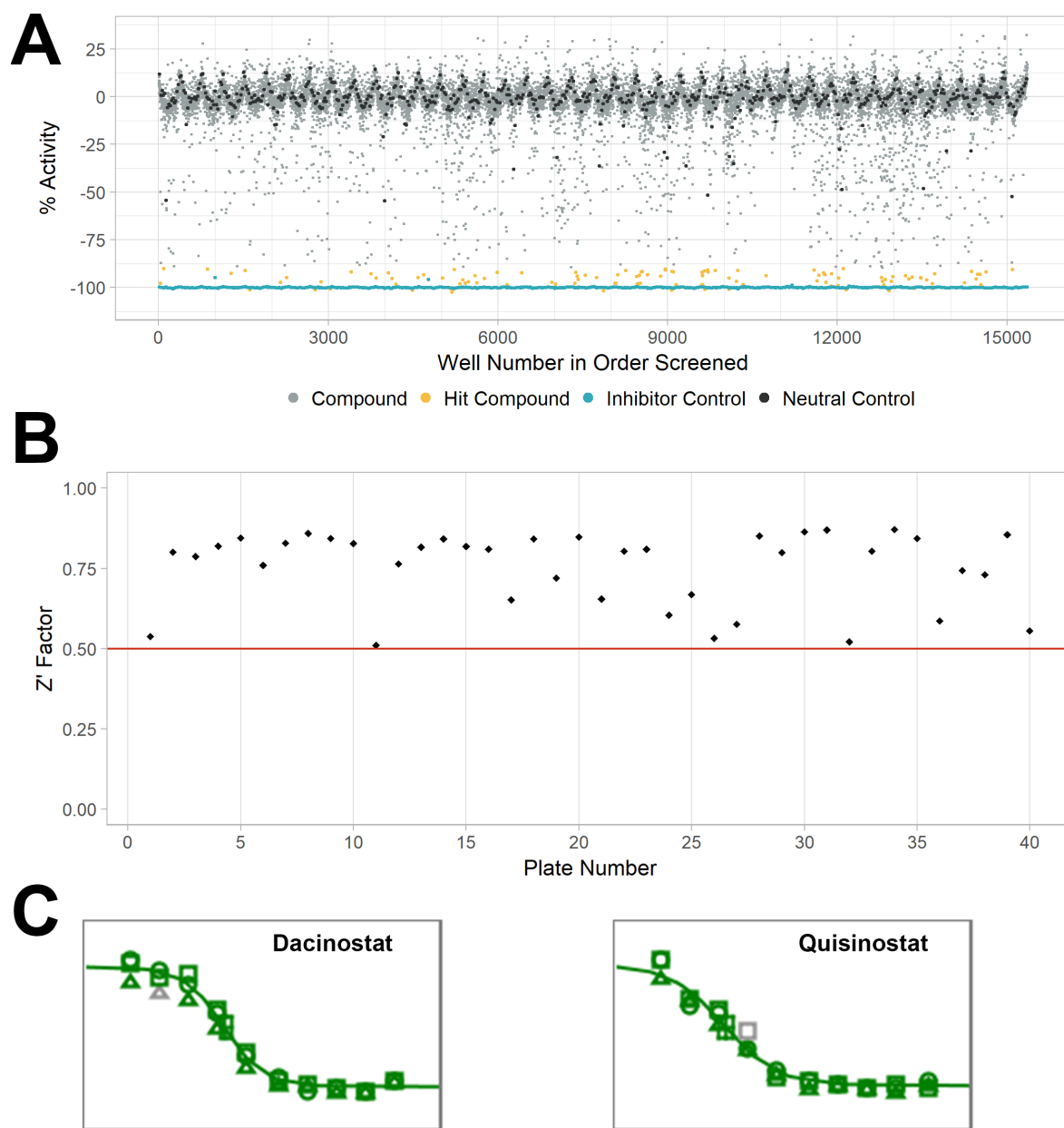

Figure S1. Compound activity and plate Z'-factor for the primary screening.

Supplement: Supplementary file 2 — Additional file 2: Figure S1. Compound activity and plate Z′-factor for the primary screening. (A) Compound activity distribution. Blue dots (Inhibitor Control): positive control group (10 μM staurosporine, 100% inhibition); black dots (Neutral Control): negative control group (DMSO group, 0% inhibition); yellow dots (Hit Compound): primary hits (hit compound) (125 compounds that displayed ≥ 90% inhibition chosen for a dose-response analysis); grey dots (Compound): compounds not chosen for further confirmation (% inhibition < 90%). Y-axis: % Activity is assessed as the percentage of viability inhibited. The activity from 12,800 library compounds and control compounds in each plate were shown. (B) Z′-factor calculated for each plate. (C) The dose response curves for cell viability of Daoy cells. Ten concentrations, 1:3 serially diluted ranging from 50 to 0.0025 μM, were used in triplicate. [file 40164_2019_153_MOESM2_ESM.pdf]
